# Wearables-based walking program in addition to usual physiotherapy care for the management of patients with low back pain at medium or high risk of chronicity: A pilot randomized controlled trial

**DOI:** 10.1371/journal.pone.0256459

**Published:** 2021-08-26

**Authors:** Hosam Alzahrani, Martin Mackey, Emmanuel Stamatakis, Debra Shirley

**Affiliations:** 1 Department of Physical Therapy, College of Applied Medical Sciences, Taif University, Taif, Saudi Arabia; 2 School of Health Sciences, Faculty of Medicine and Health, The University of Sydney, Sydney, NSW, Australia; 3 Charles Perkins Centre, School of Health Sciences, Faculty of Medicine and Health, The University of Sydney, Sydney, NSW, Australia; 4 Discipline of Physiotherapy, School of Health Sciences, Faculty of Medicine and Health, The University of Sydney, Sydney, NSW, Australia; Prince Sattam Bin Abdulaziz University, College of Applied Medical Sciences, SAUDI ARABIA

## Abstract

**Background:**

Although chronic low back pain (LBP) is a leading cause of disability and accounts for large costs, none of the available conventional treatments are clearly more favourable in treating people at increased risk of chronicity.

**Objectives:**

To examine the feasibility and initial efficacy of a wearables-based walking intervention in addition to usual physiotherapy care in people with LBP at risk of chronicity.

**Methods:**

Twenty-six adult participants, diagnosed with non-specific LBP with medium or high risk of chronicity, were recruited from physiotherapy private practices. Participants were randomized into usual physiotherapy care (control, n = 14) and usual physiotherapy care plus a wearables-based walking intervention (experimental, n = 12). The intervention duration was 8 weeks. Feasibility outcomes included recruitment rate, adherence to the intervention, dropout rate, and serious adverse events reporting rate. Other outcomes included disability and pain (primary); and physical activity level, daily walking steps, depression, pain catastrophizing and fear of movement (secondary). The outcomes were assessed at baseline, post-intervention and 26 weeks post-randomization follow-up.

**Results:**

Adherence of experimental participants with the prescribed walking program was moderate. Four participants dropped out during the intervention, and no serious adverse events were reported. Participants in the experimental group showed significant improvement in pain at 26 weeks (β = -0.38; 95% confidence interval (CI) -0.66, -0.10; P = .013), compared with the control group. No between-group differences were found for disability at any time point and pain immediately post-intervention. Experimental participants demonstrated post-intervention improvement in light-intensity (β = 156.71; 95% CI 86.79, 226.64; P < .001), moderate-intensity physical activity (β = 0.46; 95% CI 0.12, 0.80; P = .012), and daily walking steps (β = 7099.13; 95% CI 4522.93, 9675.32; P < .001). Experimental participants demonstrated post-intervention increase in pain catastrophizing (β = 0.52, 95% CI 0.18, 0.86; P = .006). No between-group differences were found for pain catastrophizing at 26 weeks and other secondary outcomes.

**Conclusion:**

Usual physiotherapy care plus a wearables-based walking intervention program was safe and moderately feasible, and provided significant reduction in pain at 26 weeks as well as increasing the total volume of light- and moderate-intensity physical activity, and daily walking steps immediately post-intervention.

## Introduction

Low back pain (LBP) is a major health problem globally; it is one of the most prevalent conditions presenting to health professionals [1, 2] and is the leading cause of years lived with disability [3], imposing a great burden on individuals and communities. While only 23% of people develop chronic non-specific LBP, it accounts for the majority of LBP-related disability and costs [4, 5].

To improve the efficacy and cost-effectiveness of LBP interventions, the STarT Back Screening Tool [6] was developed and validated to stratify patients according to the presence of modifiable physical and psychosocial prognostic variables, thereby promoting targeted treatment [6]. The tool categorizes patients into three levels of risk of developing persistent disabling LBP: low risk, medium risk (which indicates the presence of physical factors and a low level of psychosocial prognostic factors), or high risk (which indicates the presence of high levels of psychosocial prognostic factors, with or without physical factors). People categorized as medium or high risk of chronicity are more disabled by their pain and more likely to have a poor outcome than those at low risk [7]. Since those at low risk have a good prognosis and the highest probability of recovering spontaneously, minimal intervention (i.e., targeting education to remain active and self-management) is recommended [8, 9]. According to current clinical guidelines, no available treatment is clearly more favourable for treating patients at higher risk of chronicity; nevertheless, most of these guidelines recommend treating those at risk with multimodal therapy including cognitive behaviour therapy, exercise therapy and educational interventions [10, 11].

The role of physical activity in improving overall health and reducing risk factors for chronic non-communicable diseases is well documented [12]. Additionally, participating in physical activity has benefits beyond health, including social and economic benefits [13]. Walking is considered one of the simplest and most preferred types of physical activity, as it is functional, safe, accessible, and cost-effective, and it does not require any special equipment [14]. Previous reviews of walking interventions showed a positive effect on LBP outcomes, although research is still limited [15–17]. To address the limitations, these studies recommended further research to investigate the appropriate amounts of walking interventions on LBP outcomes. There were also recommendations to use objective self-monitoring devices to both measure the total physical activity level performed and to encourage people to increase their daily walking steps by providing immediate feedback on their progress [16, 17].

Some factors that can influence the success of walking programs, and consequently result in poor adherence to the program, include lack of individual motivation, ineffective goal setting, or inadequate program prescription [18]. There is emerging evidence that physical activity interventions are more effective when they include technology that allows self-monitoring of target behaviours [19, 20]. Current wearable accelerometers such as the Fitbit have features that monitor daily physical activity, set goals, and provide feedback and motivational messages. Fitbit devices have been shown to accurately count steps [21, 22] and have demonstrated the potential to improve a user’s programme adherence and motivation [23]. However, to the best of our knowledge, no study has investigated the effectiveness of the Fitbit device in facilitating a walking intervention for people with LBP.

The objective of this study was to examine the feasibility and initial efficacy of a wearables-based walking intervention, in addition to usual physiotherapy care, among people with medium or high risk of developing LBP chronicity. To examine the feasibility of the wearables-based walking intervention we measured the recruitment rate, adherence to the intervention, dropout rate, and serious adverse events reporting rate. We examined the effects of the intervention using disability and pain as primary outcomes. The secondary outcomes included physical activity level, daily walking steps, depression, pain catastrophizing and fear of movement.

## Methods

### Study design

The study design was a single-blinded randomized controlled trial. This trial was developed to comply with the guidelines of the Standard Protocol Items: Recommendations for Interventional Trials (SPIRIT) and with the Consolidated Standards of Reporting Trials (CONSORT) statement on pilot trial reporting [24]. The intervention was described according to the Template for Intervention Description and Replication (TIDieR) checklist [25]. The protocol of this study was registered prospectively at the Australian New Zealand Clinical Trials Registry (registration number ACTRN12617001404314) and approved by the Human Research Ethics Committee from the University of Sydney (project number 2017/842). The full protocol of the study was published previously in a peer-reviewed journal [26]. Deviations from the study protocol are listed in S1 Table.

### Participants

Twenty-six participants were recruited from private physiotherapy practices in Sydney, Australia. The recruited participants were assessed for eligibility by one of the research team (HA) and a practice physiotherapist. The eligibility criteria for participants used in our protocol were presented in Table 1.

**Table 1:** Eligibility criteria for participants.

***Inclusion criteria***	Aged ≥ 18 years old
	Diagnosed with non-specific LBP by a physiotherapist
	Categorized as being at medium or high risk of chronicity using the STarT Back Screening Tool [6]
	Classified as insufficiently physically active (those who engage in less than 150 minutes/week of moderate intensity, or less than 75 minutes/week of vigorous intensity or an equivalent combination of the two intensities of physical activity as determined by IPAQ [27]
	Accessibility to internet
	Readiness and ability to participate in physical activity as determined by PAR-Q. Those deemed not fit to participate in physical activity by the PAR-Q or aged over 69 years, will need a clearance from their medical practitioner before engaging in the walking intervention
***Exclusion criteria***	Diagnosed with a condition (cardiovascular diseases e.g., myocardial infarction, embolism, or uncontrolled diabetes; orthopaedic impairments; balance problems) that prevent participation in physical exercise
	Diagnosed with serious spinal pathologies (e.g., fractures, tumours or inflammatory diseases such as ankylosing spondylitis)
	Diagnosed with neurological compromise (e.g., spinal nerve compromise or cauda equina syndrome)
	Pregnancy

LBP, low back pain; IPAQ, International Physical Activity Questionnaire; PAR-Q, Physical Activity Readiness Questionnaire

### Procedure

After confirmation of eligibility and signed informed consent was obtained, baseline outcome measurements were collected. Participants were then randomly allocated to an experimental group (usual physiotherapy care plus a wearables-based walking intervention), or to a control group (usual physiotherapy care alone). The randomization process was performed previously by an independent researcher who was not involved in this study using computer-generated random numbers. Sealed envelopes were used to conceal the allocation sequence from the investigator screening participants for inclusion. Stratified block randomization was used to ensure fidelity and balance of the usual physiotherapy care given to participants in both groups at each participating clinic. The outcome measurements were administered by a researcher (HA) who guided participants through the whole study period.

### Interventions

All participants in this study received usual physiotherapy care which was provided by registered physiotherapists. The physiotherapy management provided was at the choice of the physiotherapists who determined the treatment pragmatically based on clinical reasoning. The usual physiotherapy care was a combination of, for example, strengthening exercises, stabilisation exercises, home exercises, manual therapy and education.

Participants allocated to the control group received eight weeks of usual physiotherapy care (as described above). They were also given instructions to maintain their usual physical activity level during the treatment period.

Participants randomized to the experimental group received an eight-week wearables-based walking intervention provided by one of the researchers (HA) in addition to the usual physiotherapy care. The wearables-based walking intervention was developed for patients with LBP, using physical activity guidelines [28, 29] in order to assist them maintain their usual activities, and to improve their adherence with the prescribed walking intervention. The intervention consisted of: 1) wearable device, 2) access to the 10,000 Steps website (www.10000steps.org.au) [30], and 3) progressive walking program.

During baseline measurement, all participants were also required to wear a wearable device (Fitbit Flex) for seven continuous days, to measure the total number of habitual walking steps per week [31]. Participants were not given any access to the number of walking steps attained during the baseline measurement. After conducting the randomization process, the baseline average daily walking steps (measured by Fitbit) for participants allocated in the experimental group were calculated by dividing the total weekly number of walking steps by seven (total walking steps per week/7). In the first week of the intervention the target for each participant was ‘the average daily walking steps plus 10%’, and each week the target of the previous week was progressed by a further 10%. To comply with current physical activity guidelines, participants were also asked to walk the prescribed number of steps at a moderate intensity (i.e., brisk walking, 100 steps/min) [32–35]. Participants were asked to carry out the prescribed walking program on at least five days per week for eight weeks.

Participants randomized to the experimental group were given the Fitbit wearable device and attended a training session delivered by the same researcher (HA). The training session involved installing and setting up the Fitbit wearable device and account, registering the participant in the 10,000 Steps website and synchronising the device with the Fitbit account. Participants were also provided with instructions of using the device and account including monitoring step counts as well as other features. Participants were instructed to wear the Fitbit device continuously during the day, even during showering or while engaging in any water-based activities, and take it off while sleeping.

The Fitbit is a waterproof monitoring device that can be worn as a wristband for 24 hours, which can be more convenient for participants than other devices worn on the waist. The Fitbit contains a tri-axial accelerometer, which has been shown to be accurate and valid in measuring physical activity and quantifying steps [21, 22, 36]. The fully charged Fitbit battery lasts for approximately 5 days (detailed information on the Fitbit Flex device can be found on https://www.fitbit.com/global/us/home). The Fitbit wearable device enabled participants to monitor their progress in meeting the target number of steps each day. Further, the Fitbit wearable device acted as a motivational feedback tool providing information via the Fitbit application on the number of walking steps attained. The 10,000 Steps website is a non-profit promotion web-based platform that was developed by Central Queensland University in Australia to increase the physical activity level of the general community and it can automatically connect and synchronise with the Fitbit account. We used this website to encourage participants to track and monitor progress in achieving the daily steps target, use the Fitbit device and share their progress with other participants. Also, it enabled the investigator to monitor and track the participants’ progress and keep in contact with them. The website has high levels of usability. Further details about the conceptual basis for wearables-based Walking Intervention and development can be found elsewhere [26].

### Outcomes

The protocol feasibility was evaluated by measuring the recruitment rate, dropout rate, participant’s adherence with the walking intervention, and occurrence of serious adverse events. The adherence with the walking intervention was measured using the Fitbit wearable device during the treatment period (8 weeks) by assessing the number of steps attained each day. Adherence with the prescribed walking intervention was calculated by dividing the average attained walking steps per day by the average target walking steps in that week and expressing it as a percentage.

All primary and secondary outcomes were measured at baseline (week 0), post-intervention (week 9) and post-randomization follow-up (week 26).

The primary outcomes were disability and pain. Disability was measured by the 10-item modified Oswestry Disability Index (ODI), with higher scores indicating greater disability (range, 0–100) [37]. The ODI has demonstrated a good validity, reliability and responsiveness for detecting change in disability in people with LBP [37, 38]. The worst pain intensity was measured in the past 24 hours by the Visual Analogue Scale (VAS), on scale from 0 (no pain) to 10 (worst pain) [39]. The VAS is a valid and reliable tool for assessing pain in participants with LBP [40].

Secondary outcomes included measures of physical activity intensity (light-, moderate-, and vigorous-intensity) and walking steps as well as depression, pain catastrophizing and fear of movement. Physical activity level and walking steps were measured during waking hours over one week with the Axivity AX3 (Newcastle upon Tyne, UK; product website: http://axivity.com/product/ax3), worn on non-dominant wrist. The Axivity has been shown to be accurate and valid in assessing physical activity [41, 42] and measuring walking steps [43–45]. Depression was measured by the 21-item Beck Depression Inventory (BDI) [46], which has a good internal consistency and good content validity [47]. Participants’ thoughts and feelings about pain were measured by the 13-item Pain Catastrophizing Scale (PCS) [48], which has been shown to be reliable and valid [49, 50]. Fear of movement was measured by the 17-item Tampa Scale for Kinesiophobia (TSK); this tool has good validity and reliability in assessment the fear of movement resulting from feeling of vulnerability to re-injury in participants with pain [51, 52].

The subjective data (disability, pain, depression, pain catastrophizing and fear of movement) were collected and administered using Research Electronic Data Capture (REDCap) [53]. Further details concerning the outcomes can be found in the study protocol [26].

### Axivity AX3 data extraction

The Axivity was set up to capture data for seven continuous days at 100Hz with a dynamic range of +-8g. Physical activity intensity levels (light, moderate and vigorous) were extracted from the Axivity device using OMGUI software (GITHub, University of Newcastle) and the standard thresholds were used to aggregate data into light (≥1.5 metabolic equivalent (MET), <4 MET), moderate (≥4 MET, <7 MET), and vigorous (≥7 MET) activity [54]. The walking bouts were extracted using MATLAB program (version R2018a) with threshold of 60s. The average number of total steps per day was calculated by summing up the number of steps in that week and then dividing the total number of steps by seven [55]. The algorithm used by MATLAB program is based on a method presented in previous studies [56]. For a day to be ‘valid’ for inclusion in the analyses, participants had to have worn the Axivity device for at least 8 hours. The wear time estimation was extracted using OMGUI. The algorithm used by OMGUI is based on a method proposed in a previous study [57]. All participants with at least one valid day of Axivity wear have been included in the analyses. However, the majority of participants (baseline: 99%, post-intervention: 100%, 26 weeks: 77%) had at least 3 valid days of wear.

### Sample size

Although a pilot RCT does not require estimating the sample size [58], a minimum of 12 participants per group is recommended to ensure scientific validity of the pilot trial results [59, 60]. Therefore, the present pilot RCT would need a minimum of 24 participants with 12 participants per group. Furthermore, the results of this pilot trial provide information on the mean difference between groups and standard deviation (SD) necessary for use in the calculation of the sample size for future fully powered trial.

### Statistical analysis

Demographic variables, clinical characteristics and outcome measures were displayed by means (standard deviation (SD)) or median (interquartile range (IQR)). The treatment effects were examined using linear regression. Assumptions of linear regression were assessed and met. The normality of outcomes was examined using Shapiro-Wilk test, and the variables that were not normally distributed were log transformed. The statistical analysis followed the intention-to-treat principle in which all participants were analyzed in the groups to which they were randomized, regardless of whether they withdrew from their allocation [61]. Missing data were replaced with the mean value for each item [62]. The analysis was conducted in consultation with an expert statistician who was blinded to the treatment allocation. Data were analyzed using IBM Statistical Package for Social Sciences (SPSS) version 22 [63].

## Results

### Flow of participants through the study

**Fig 1:**
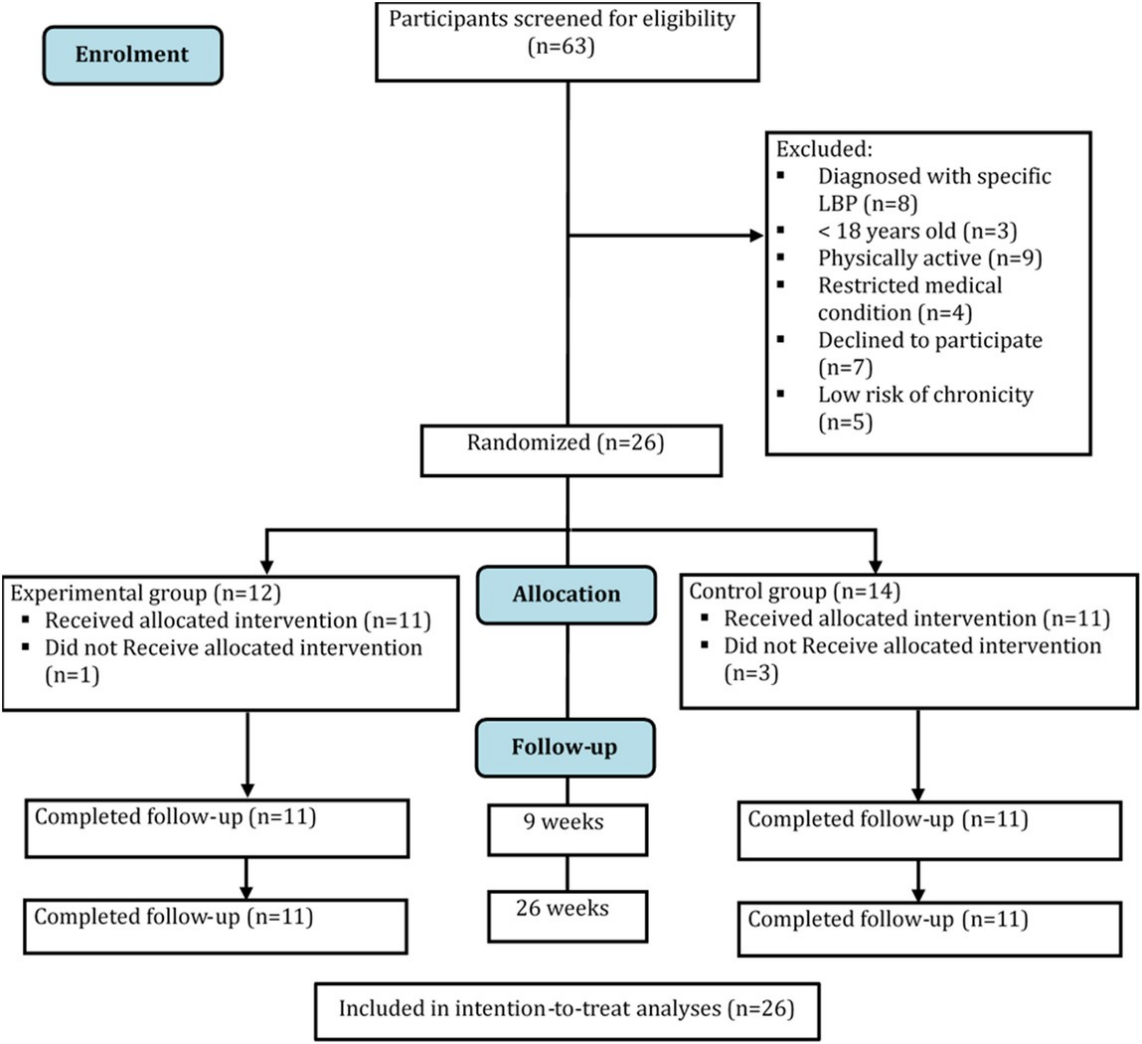
Flow of participants through the trial.

A total of 63 participants with medium or high risk of chronicity of LBP were screened for inclusion between December 2017 and November 2018. Twenty-six participants were eligible to be included in the study. Twenty-two participants have completed the study and four participants (1 from the experimental group and 3 from the control group) dropped out during the intervention period: two for personal reasons and two were not interested in completing the study. Flow of participants through the study is shown in Fig 1.

### Characteristics of participants

Twenty-six participants (11 females (42.3%)) with a mean age of 43.6 years, were randomized to either the experimental group (n = 12) or control group (n = 14). Table 2 presents the baseline characteristics of participants by study arm.

**Table 2:** Participants demographics and outcome measures variables at baseline.

Variables	Total Sample Size (n = 26) ^a^	Experimental (n = 12) ^a^	Control (n = 14) ^a^
**Characteristics**			
Age (y)	43.6 (14.3)	49.0 (13.4)	39.0 (13.8)
Gender, female, no. (%)	11 (42.3)	3 (11.5)	8 (30.8)
Body mass index (kg/cm^2^)	29.38 (6.76)	29.30 (7.59)	29.44 (6.25)
Married, no. (%)	20 (76.9)	9 (34.6)	11 (42.3)
Completed university degree, no. (%)	8 (30.8)	6 (23.1)	2 (7.7)
Employed full-time, no. (%)	12 (46.2)	6 (23.1)	6 (23.1)
Currently smoking, no. (%)	5 (19.2)	1 (3.8)	4 (15.4)
**Outcomes**			
ODI score (0–100; Median (IQR))	24 (31)	17 (28)	30 (39)
VAS score (0–10; (Median (IQR))	4 (3)	4 (2)	5 (2)
Physical activity (minutes/day)			
Light	286.76 (95.07)	269.39 (104.87)	301.65 (86.93)
Moderate (Median (IQR))	76.07 (34.45)	80.93 (28.01)	68.16 (45.22)
Vigorous (Median (IQR))	0.29 (1.22)	0.36 (1.11)	0.29 (1.22)
Walking steps			
Walking steps (steps/day)	13302 (5141)	12998 (4217)	13563 (5968)
BDI score (0–63; Median (IQR))	11.00 (17)	7.50 (11)	13.50 (20)
PCS score (0–52; Median (IQR))	17.50 (24)	18.50 (24)	17 (32)
TSK score (17–68)	40.19 (9.20)	39.92 (7.79)	40.43 (10.56)

Abbreviations: BDI, Beck Depression Inventory; ODI, Oswestry Disability Index; PCS, Pain Catastrophizing Scale; SD, standard deviation; TSK, Tampa Scale for Kinesiophobia; VAS, Visual Analogue Scale; y, years.

^a^ Data are reported as mean (SD), unless otherwise indicated.

### Protocol feasibility

The trial protocol was deemed safe and moderately feasible. Adherence of participants in the experimental group with the prescribed walking program was moderate. Participants in the experimental group adhered to 67.1% of the prescribed walking program. There were no serious adverse events reported in this study. However, there was difficulty in recruiting participants as we were only able to recruit 26 participants.

### Adherence with walking intervention program

**Fig 2:**
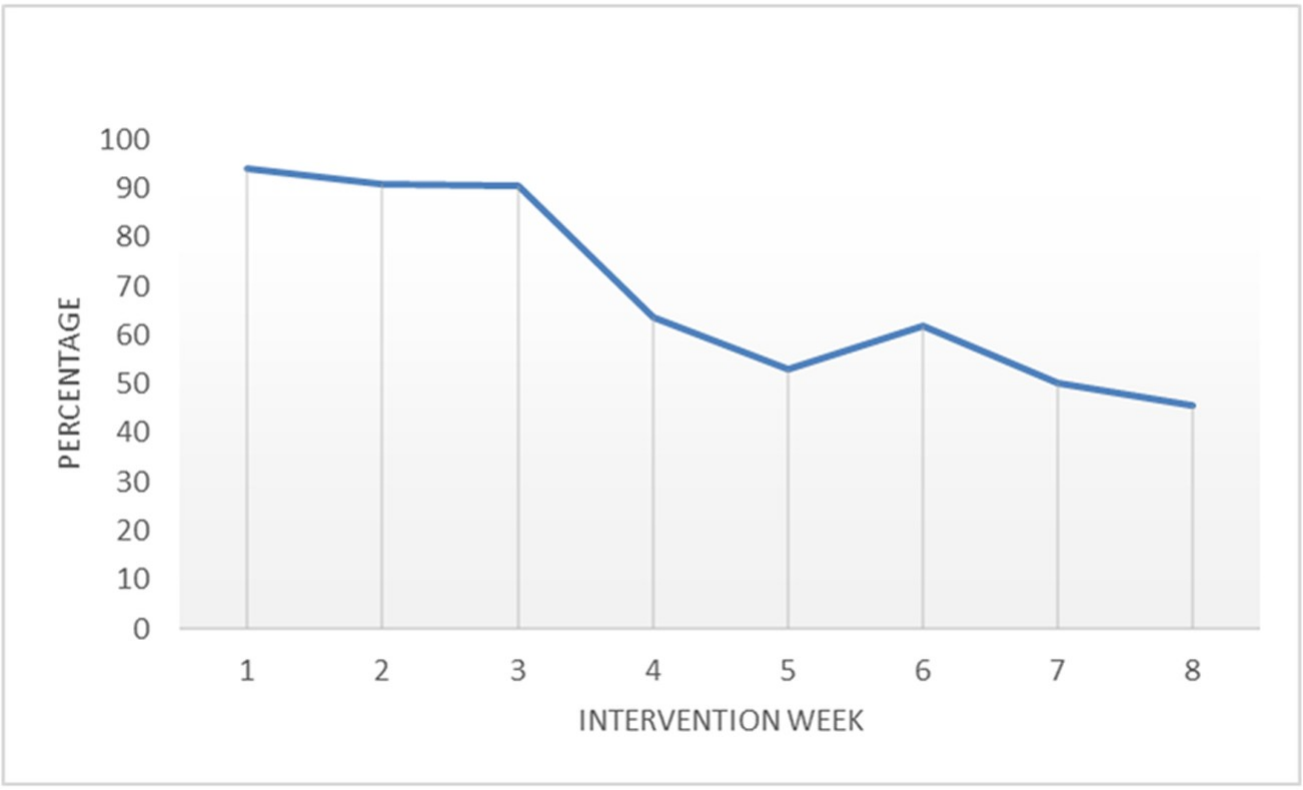
Participants’ adherence to prescribed walking program.

Participants in the experimental group (n = 11) adhered to 67.1% (range = 56.3) of the prescribed walking program. The median percentage adherence of participants to the walking program decreased by an average of 7.7% (*P*< .001) each week throughout the program. Participants adhered to 94.2% (range = 106) of the prescribed walking program in the first week followed by 90.8% (range = 109) and 90.5% (range = 141.1) in the second and third weeks, respectively. The median percentage adherence started to decline from the fourth week until it reached 45.7% (range = 82.2) in the last week of the program (Fig 2).

The correlation of outcomes with the participants’ adherence to the prescribed walking program is displayed in S2 Table. There was a strong, inverse correlation between treatment adherence and pain intensity at 26 weeks follow-up (*r* = -0.665; n = 11; *P* = .026), but not with the other outcomes.

### Primary outcomes

There were no between-group differences in disability post-intervention (*β* = -0.05, 95% CI -0.30, 0.20; *P* = .670) or at 26 weeks (*β* = -0.15, 95% CI -0.52, 0.21; *P* = .374), or in pain post-intervention (*β* = -0.11, 95% CI -0.33, 0.10; *P* = .273). However, participants in the experimental group had significantly lower pain at 26 weeks (*β* = -0.38, 95% CI -0.66, -0.10; *P* = .013), compared with the control group. Table 3 shows the mean or median scores and differences between groups post-intervention and at 26 weeks for all outcomes.

**Table 3:** Scores for primary and secondary outcomes and differences between groups.

Outcomes	Experimental (n = 12)	Control (n = 14)	Unadjusted Between-Group Difference			Adjusted Between-Group Difference ^d^		
	Mean (SD) ^**a**^	Mean (SD) ^**a**^	β	95% CI	*P*	β	95% CI	*P*
**Primary Outcomes**								
Disability (ODI score (0–100))^b^^,^ ^c^								
Baseline	17 (28)	30 (39)						
9 weeks	14 (13)	19.55 (11)	-0.07	-0.23, 0.09	0.352	-0.05	-0.30, 0.20	0.670
26 weeks	12.64 (11)	19.45 (11)	-0.23	-0.48, 0.03	0.075	-0.15	-0.52, 0.21	0.374
Pain (VAS score (0–10))^b^^,^ ^c^								
Baseline	4 (2)	5 (2)						
9 weeks	3 (3)	3 (1)	-0.01	-0.21, 0.19	0.944	-0.11	-0.33, 0.10	0.273
26 weeks	1 (2)	3.36 (4)	-0.28	-0.54, -0.03	0.033 *	-0.38	-0.66, -0.10	0.013 *
**Secondary Outcomes**								
Physical activity (minutes/day)								
Light								
Baseline	269.39 (104.87)	301.65 (86.93)						
9 weeks	314.77 (137.68)	244.24 (126.29)	70.53	-36.35, 177.41	0.186	156.71	86.79, 226.64	<0.001 **
26 weeks	269.76 (110.75)	271.48 (116.85)	-1.72	-94.35, 90.92	0.970	45.48	-55.10, 146.06	0.350
Moderate^b^^,^ ^c^								
Baseline	80.93 (28.01)	68.16 (45.22)						
9 weeks	103.13 (46.49)	84.95 (77.48)	0.18	-0.14, 0.49	0.250	0.46	0.12, 0.80	0.012 *
26 weeks	84.41 (27.25)	76.09 (50.81)	0.17	-0.10, 0.45	0.205	0.30	-0.05, 0.65	0.086
Vigorous^b^^,^ ^c^								
Baseline	0.36 (1.11)	0.29 (1.22)						
9 weeks	1 (1.54)	0.84 (1.42)	0.06	-0.26, 0.39	0.683	0.06	-0.40, 0.52	0.778
26 weeks	1.21 (1.58)	0.95 (2.01)	-0.09	-0.60, 0.43	0.735	0.15	-0.44, 0.73	0.573
Walking steps (steps/day)								
Baseline	12998.30 (4217.78)	13563.10 (5968.27)						
9 weeks	15647.42 (5813.38)	11978.75 (6516.98)	3668.66	-1368.91, 8706.23	0.146	7099.13	4522.93, 9675.32	<0.001 **
26 weeks	13770.09 (5517.18)	11600.6131 (5542.77)	2169.484	-2321.37, 6660.33	0.329	4582.37	-124.38, 9289.12	0.056
Depression (BDI score (0–63))^b^^,^ ^c^								
Baseline	7.50 (11)	13.50 (20)						
9 weeks	8.59 (11)	8.36 (10)	0.01	-0.36, 0.38	0.959	0.11	-0.34, 0.56	0.616
26 weeks	6.37 (6)	10.32 (7)	-0.23	-0.50, 0.04	0.094	-0.19	-0.54, 0.15	0.243
Pain Catastrophizing (PCS score (0–52))^b^^,^ ^c^								
Baseline	18.50 (24)	17 (32)						
9 weeks	13 (11)	9.50 (10)	0.23	-0.14, 0.59	0.208	0.52	0.18, 0.86	0.006 *
26 weeks	14.55 (21)	10.91 (21)	0.10	-0.35, 0.55	0.654	0.32	-0.14, 0.78	0.151
Kinesiophobia (TSK score (17–68))								
Baseline	39.92 (7.79)	40.43 (10.56)						
9 weeks	40.82 (8.88)	37.09 (7.30)	3.73	-2.82, 10.27	0.251	2.22	-3.98, 8.42	0.457
26 weeks	43.18 (8.49)	36.36 (8.41)	6.82	-0.04, 13.68	0.051	6.15	-2.07, 14.37	0.131

*p < 0.05,

**p < 0.001

Abbreviations: BDI, Beck Depression Inventory; IQR, interquartile range; ODI, Oswestry Disability Index; PCS, Pain Catastrophizing Scale; SD, standard deviation; TSK, Tampa Scale for Kinesiophobia; VAS, Visual Analogue Scale; y, years.

^a^ Data are reported as mean (SD), unless otherwise indicated.

^b^ Data are reported as median (IQR).

^c^ Variable was log transformed as their distribution deviated from normal.

^d^Adjusted for baseline values of primary and secondary outcomes.

Note: There were 12, 11, and 11 participants in the experimental group with available data at baseline, 9-weeks, and 26-weeks follow-ups, respectively. There were 14, 11, and 11 participants in the control group with available data at baseline, 9-weeks, and 26-weeks follow-ups, respectively.

### Secondary outcomes

Participants in the experimental group demonstrated improvement in light-intensity physical activity post-intervention (*β* = 156.71, 95% CI 86.79, 226.64; *P*< .001) and moderate-intensity physical activity post-intervention (*β* = 0.46, 95% CI 0.12, 0.80; *P* = .012), compared with the control group. However, these improvements were no longer statistically significant at 26 weeks. We found no significant differences between groups in vigorous-intensity at any time point. Participants in the experimental group demonstrated a significant increase in number of daily walking steps post-intervention (*β* = 7099.13, 95% CI 4522.93, 9675.32; *P*< .001) but not at 26 weeks, compared with the control group (Table 3).

Participants in the experimental group had significantly higher pain catastrophizing post-intervention (β = 0.52, 95% CI 0.18, 0.86; P = .006), compared with the control group. However, there were no differences between groups for pain catastrophizing at 26 weeks and the other secondary outcomes of depression, and fear of movement at any time point (Table 3).

## Discussion

This study found that the addition of a wearables-based walking intervention to usual physiotherapy care was safe and moderately feasible, as well as effective in improving pain at 26 weeks, and increasing light- and moderate-intensity physical activity and daily walking steps immediately post-intervention although these improvements were not sustained at 26 weeks. However, participants in the experimental group had significantly higher pain catastrophizing immediately post-intervention, compared with the control group. There were no between-group differences in pain catastrophizing at 26 weeks, and disability, depression, fear of movement, and vigorous-intensity physical activity at any time point. However, as this study is a pilot trial with a small sample size, no observable significant effects were expected to be seen in the outcomes measures. It should also be noted that including a relatively small sample size increased the possibility for a type 2 error.

This pilot trial showed that the prescribed walking intervention program for participants with LBP with medium or high risk of chronicity is safe and moderately feasible. We observed a moderate rate of adherence, a low dropout rate, and observed no serious adverse events. Possible reasons for dropping out the study might be due to participants lacking the desire/motivation to commit to some or all of the trial tasks, for example filling in the questionnaires and engaging in physical activity interventions. In addition, there was difficulty in recruiting participants in this study. One suggested reason for the low recruitment rate was that because this study was unfunded and we were unable to pay for the overall treatment sessions and travelling costs.

The significant reduction in pain might be explained by the increase in physical activity level and walking steps among participants in the experimental group, where the estimated increases in daily walking steps from baseline was 2649 steps (20%) post-intervention and 772 steps (6%) at 26 weeks. This finding is consistent with the study published by McDonough, Tully [55] which found that participants who received pedometer driven walking intervention increased their daily walking steps at eight weeks by 2776 from baseline and had a greater improvement in disability and pain at 24 weeks, compared with those who received education/advice only. Furthermore, the study published by Krein, Kadri [64] found an increase of approximately 700 daily walking steps (measured by a pedometer) and reduction in pain at 24 weeks in the intervention participants. The delay in the effects of the experimental intervention in reduction of pain remain to be investigated; however, participants in the experimental group had increase in pain catastrophizing immediately post-intervention which might delay the recovery [65]. Various studies showed that pain catastrophizing is associated with more intense experience of pain and poor treatment outcomes [66–68]. Thus, including additional interventions (e.g., cognitive behavioural intervention) to address the negative perceptions of pain (pain catastrophizing) can help in avoiding delayed recovery and chronicity [65, 69]. To our knowledge, there is no study investigated the effectiveness of physical activity interventions in reducing pain catastrophizing in people with chronic LBP. Therefore, a future trial investigating a combined wearables-based walking program and cognitive behavioural intervention in management people with medium or high risk of LBP chronicity is required.

Although the wearables-based walking intervention program was effective in increasing physical activity levels immediately post-intervention, there was no effect on disability, depression and fear of movement at any time point. This finding was inconsistent with a previous trial by Krein, Kadri [64] which found that the internet-mediated walking intervention was effective in reducing disability at 24 weeks. However, the participants included in that study were classified as inactive at baseline (mean< 5000 steps/day, measured by pedometer) while participants included in our study were classified as active at baseline (mean> 10,000 steps/day, measured by Axivity), suggesting there was a possibility of ceiling effect. Therefore, inactive people might be more likely to show improvements in physical activity levels and walking steps. A future trial investigating the effectiveness of the wearables-based walking intervention program in inactive people is recommended.

With the proven benefits of physical activity for overall health [12, 17], the focus of this intervention was on increasing amount and intensity of habitual physical activity. The wearables-based walking intervention program was effective in motivating participants in the experimental group and increasing their physical activity level. This result is important as the existing literature indicated the role of physical activity in reducing the risk of progression to chronicity in LBP [70, 71]. The average daily walking steps increased significantly in the experimental group post-intervention, compared with the control group. Estimated increase in daily walking steps in the experimental group from baseline to post-intervention was 2649 steps. In contrast, the estimated decrease in daily walking steps in the control group from baseline to post-intervention was 1585 steps. Additionally, the light- and moderate-intensity physical activity increased significantly in the experimental group post-intervention compared with the control group, with an adjusted between-group difference of 157 minutes/day and 43 minutes/day, respectively. Interestingly, there was no between-group difference in their level of vigorous-intensity physical activity. One explanation of that is might be because of the focus of the intervention program on moderate-intensity physical activity and walking steps.

We found a strong, negative correlation between intervention’s adherence and pain intensity at 26 weeks follow-up. This finding indicates that improving participant’s adherence to the intervention is likely to affect the success of the intervention program. The walking intervention program was moderately feasible in improving adherence with the prescribed walking intervention program. Participants in the experimental group adhered to 67.1% of the prescribed walking intervention. However, the median percentage adherence declined gradually from the fourth week until reached 45.7% in the last week of the program. This finding was in agreement with a previous study which found that the mean percentage adherence (measured by a pedometer) of chronic LBP participants with the 8-week progressive walking program was 70% [55]. Consistent adherence to treatment can be challenging, suggesting that additional strategies to promote adherence can help to maintain consistent engagement with the intervention program. One strategy is using new models of trackers with screens allowing participants to monitor their progress in real time using the watch without the need to go to the application.

We also observed that participants preferred to monitor their physical activity progress using the mobile application rather than using the website. Participants used the Fitbit application to monitor their physical activity progress more often than the 10,000 steps website. Therefore, the influence of the website in motivating participants to monitor their progress and adherence to the treatment might be limited. That might be one of the potential explanations for the gradually decrease in adherence to intervention during the eight-week intervention program (measured using Fitbit).

All participants were instructed to wear the Fitbit for the first week and the Axivity for three separate weeks during the study. This may increase the burden on participants, and consequently increase the chance of them dropping out of study. Furthermore, participants randomized to the experimental group were also instructed to wear the Fitbit for two months during the intervention period. Therefore, to decrease the burden on participants and for their comfort during the study, they were instructed to place it on their wrist. However, placing the wearable devices (Axivity and Fitbit) on the wrist may have resulted in more movement being detected during upper arm movement when the wrist is involved, than during walking [72].

This study has several strengths which include: 1) measuring the change in physical activity level and daily walking steps for participants objectively using the Axivity device; 2) prescribing a progressive walking program that is tailored to each participant with the aim of motivating participants and encouraging them to increase their activity level; and 3) measuring the participants’ adherence to the intervention objectively using the Fitbit device.

This study had several methodological limitations. First, participants met the inclusion criteria for the study if they were classified as inactive using the IPAQ as an initial screen for level of physical activity. As a self-report questionnaire, the IPAQ is likely to be subject to recall bias, as none of the included participants were classified as inactive at baseline when measured by the accelerometer. Therefore, using accelerometer as an initial screen is recommended in future research. Second, our sample size was relatively small and underpowered to detect significant improvements in our specified outcomes. Third, self-selection may be a factor in that people who already physically active may have been more likely to volunteer for the trial. Fourth, overweight and/or obese participants were not excluded from this study, which might have a significant impact on the outcomes of low back pain and physical activity.

## Conclusion

Our study has shown the efficacy of the addition of the wearables-based walking intervention to usual physiotherapy care in management participants with medium or high risk of LBP chronicity. Furthermore, a wearables-based walking program is moderately feasible and safe to carry out in individuals with LBP with medium or high risk of chronicity. In addition, participants in the experimental group demonstrated improvements in pain at 26 weeks, as well as greater increases in light- and moderate-intensity physical activity and walking steps immediately post-intervention. These preliminary findings suggest that physical activity may play an important role in alleviating some of the consequences of chronic LBP. However, there was no effect on pain catastrophizing at 26 weeks, and disability, depression, fear of movement, and vigorous-intensity physical activity at any time point.

## Supporting information

S1 Table Deviations from the study protocol. (DOCX)

S2 Table Bivariate correlation of outcomes with participants adherence to the prescribed walking program (n = 11). (DOCX)

S1 Protocol (DOCX)

S1 Checklist CONSORT checklist. (DOC)

S1 File Dataset. (XLSX)
