# Supplementary material for: Wearables-based walking program in addition to usual physiotherapy care for the management of patients with low back pain at medium or high risk of chronicity: A pilot randomized controlled trial
Source: PLoS One. 2021 Aug 26;16(8):e0256459. doi: 10.1371/journal.pone.0256459 (PMC8389429; doi:10.1371/journal.pone.0256459)
Supplement: S1 Table — (DOCX) [file pone.0256459.s001.docx]

**S1 Table. Deviations from the study protocol.**

| **Protocol method** | **Deviation from protocol method, with justification** |
| --- | --- |
| We planned to conduct a full-scale trial (see the protocol). | The design of this study was changed to a “pilot trial” to determine the initial efficacy, safety and feasibility of a full-scale trial.  The following changes were conducted accordingly in the study:   1. Additional outcomes were included in the pilot trial to determine the feasibility of a full-scale trial including recruitment rate, dropout rate, participant’s adherence with the walking intervention, and occurrence of serious adverse events. 2. The sample size calculations were changed in consultation with an experienced statistician. Therefore, the present pilot RCT would need a minimum of 24 participants with 12 participants per group, as it is recommended (1, 2). 3. The statistical analyses were changed in consultation with an experienced statistician. The edited statistical section read in the text as: “Demographic variables, clinical characteristics and outcome measures were displayed by means (standard deviation (SD)) or median (interquartile range (IQR)). The treatment effects were examined using linear regression. Assumptions of linear regression were assessed and met. The normality of outcomes was examined using Shapiro-Wilk test, and the variables that were not normally distributed were log transformed. The statistical analysis followed the intention-to-treat principle in which all participants were analyzed in the groups to which they were randomized, regardless of whether they withdrew from their allocation [62]. Missing data were replaced with the mean value for each item [63]. The analysis was conducted in consultation with an expert statistician who was blinded to the treatment allocation. Data were analyzed using IBM Statistical Package for Social Sciences (SPSS) version 22 [61].” |

1. Julious SA. Sample size of 12 per group rule of thumb for a pilot study. Pharmaceutical Statistics: The Journal of Applied Statistics in the Pharmaceutical Industry. 2005;4(4):287-91.

2. Kieser M, Wassmer G. On the Use of the Upper Confidence Limit for the Variance from a Pilot Sample for Sample Size Determination. Biometrical Journal. 1996;38(8):941-9.
