# Supplementary material for: Wearables-based walking program in addition to usual physiotherapy care for the management of patients with low back pain at medium or high risk of chronicity: A pilot randomized controlled trial
Source: PLoS One. 2021 Aug 26;16(8):e0256459. doi: 10.1371/journal.pone.0256459 (PMC8389429; doi:10.1371/journal.pone.0256459)
Supplement: S2 Table — (DOCX) [file pone.0256459.s002.docx]

S2 Table. Bivariate correlation of outcomes with participants adherence to the prescribed walking program (n = 11).

| Outcomes | *r* | *p* |
| --- | --- | --- |
| Primary Outcomes |  |  |
| Disability (ODI score (0-100)) |  |  |
| 9 weeks | .092 | .789 |
| 26 weeks | .134 | .695 |
| Pain (VAS score (0-10)) |  |  |
| 9 weeks | -.024 | .944 |
| 26 weeks | -.665 | .026* |
| Secondary Outcomes |  |  |
| Physical activity (minutes/day) |  |  |
| Light |  |  |
| 9 weeks | -.100 | .770 |
| 26 weeks | -.078 | .819 |
| Moderate |  |  |
| 9 weeks | -.356 | .283 |
| 26 weeks | .110 | .748 |
| Vigorous |  |  |
| 9 weeks | -.241 | .476 |
| 26 weeks | -.403 | .220 |
| Walking steps (steps/day) |  |  |
| 9 weeks | -.182 | .593 |
| 26 weeks | -.054 | .874 |
| Depression (BDI score (0-63)) |  |  |
| 9 weeks | -.508 | .110 |
| 26 weeks | -.100 | .770 |
| Pain Catastrophizing (PCS score (0-52)) |  |  |
| 9 weeks | -.315 | .345 |
| 26 weeks | -.382 | .247 |
| Kinesiophobia (TSK score (17-68)) |  |  |
| 9 weeks | -.008 | .981 |
| 26 weeks | .080 | .814 |
| **p* < 0.05  Abbreviations: BDI, Beck Depression Inventory; ODI, Oswestry Disability Index; PCS, Pain Catastrophizing Scale; TSK, Tampa Scale for Kinesiophobia; VAS, Visual Analogue Scale. | | |
