# Supplementary material for: Wearables-based walking program in addition to usual physiotherapy care for the management of patients with low back pain at medium or high risk of chronicity: A pilot randomized controlled trial
Source: PLoS One. 2021 Aug 26;16(8):e0256459. doi: 10.1371/journal.pone.0256459 (PMC8389429; doi:10.1371/journal.pone.0256459)
Supplement: S1 Protocol — (DOCX) [file pone.0256459.s003.docx]

**TITLE**

Accelerometer-based facilitated walking program in addition to usual care for the management of patients with low back pain at medium or high risk of chronicity: a protocol for a randomised controlled trial

**ABSTRACT**

**Objectives:** To examine the efficacy of Fitbit facilitated walking intervention in addition to usual care in people with low back pain who have been categorized as being at medium or high risk of chronicity in reducing disability and pain.

**Design:** Randomised controlled trial**.**

**Setting:** Physiotherapy outpatient departments of public and private hospitals or private physiotherapy practices in Sydney, Australia.

**Participants:** This study will recruit participants who are aged 18 years or over, have been diagnosed with non-specific low back pain with medium or high risk of chronicity and classified as physically inactive.

**Interventions:** Participants will be randomised into two groups: usual physiotherapy care (n=34) and usual physiotherapy care plus Fitbit facilitated walking intervention program (n=34). The treatment duration will be 8 weeks. The outcomes will be assessed at baseline, post-intervention and 26 weeks post randomisation follow-up.

**Main outcome measures:** The primary outcomes for this study are disability and pain. The secondary outcomes include physical activity level and walking steps, depression, fear of movement and pain catastrophising,

**Discussion:** The results of this study will provide empirical evidence on the efficacy of Fitbit facilitated walking intervention program, when added to usual care, in a clinical setting for improving disability and pain, and other clinical outcomes in people with low back pain.

**Trial registration:**

**Keywords:** Low back pain, Physical activity, Walking, Fitbit, Randomised controlled trial, Protocol.

**INTRODUCTION**

Low back pain (LBP) is one of the most common conditions presenting to health professional and is a major health problem globally.^1^ The lifetime prevalence of LBP is reported to be as high as 84% worldwide.^2^ Around 23% of the population has chronic non-specific LBP.^3, 4^ The risk of developing chronic LBP has been associated with various individual, psychosocial (e.g. depression, fear of movement and pain catastrophizing) and occupational factors ^5-11^; leading to high levels of disability and imposing high direct and indirect costs to the individual and the community.^1-3, 6, 8-10, 12^

Several studies have shown the association of catastrophizing with depression and fear of movement.^13-15^ According to the Fear Avoidance Model, people with chronic LBP presenting with higher levels of fear of movement are more probably to develop avoidance behaviour, leading to greater disability and lower physical activity levels.^16-18^ A recent meta-analysis showed that people with chronic LBP with high levels of disability are likely to present with lower levels of physical activity.^19^ Promoting a physically active lifestyle might therefore improve the psychosocial factors and reduce disability. Additionally, encouraging people to participate in physical activity has benefits beyond health, including social and economic benefits. The health benefits of physical activity in improving overall health status and reducing risk factors for non-communicable diseases are well documented.^20^

Walking is one of the simplest and preferred types of physical activity as it is functional, safe, accessible, cost-effective and does not require any special equipment.^21^ Despite the potential health benefits of walking, few studies have investigated its effectiveness in people with non-specific LBP. There is however conflicting results found in these studies. One trial^22^ found that a walking intervention was effective in decreasing disability and pain in participants with chronic LBP. But another trial^23^ reported a significant reduction in pain and disability in favor of other group with conventional physiotherapy and a group with medical exercise therapy compared to walking; this trial prescribed the walking intervention to participants as instructions and to be performed individually, and they were not supervised or monitored. However, the intensity and duration of walking were not measured, and the patient’s adherence to the treatment was not reported. Therefore, more high quality evidence on the effectiveness of walking for LBP is needed to guide prescription of walking as an intervention.

Lack of compliance is one of the challenges which may affect the success of walking programs.^24^ Poor compliance may be due to lack of individual motivation, goal setting, or inadequate program prescription. There is emerging evidence that physical activity interventions are more effective when they include technology that allows self-monitoring of target behaviour.^25, 26^ Current wearable accelerometers such as the Fitbit devices have features such as monitoring daily physical activity, providing feedback and motivating messages, and setting up goals. The wearable Fitbit devices have not only shown accuracy and validity in measuring step counts but have also shown potential to improve a person’s adherence and motivation.^27-29^ However, to the best of our knowledge no study has previously investigated the effect of the Fitbit device as a facilitator of a walking intervention in addition to usual care for the management of people with LBP.

**OBJECTIVES**

1. To examine the efficacy of Fitbit facilitated walking intervention in people with LBP with medium or high risk of chronicity in reducing disability and pain.

2. To examine the efficacy of Fitbit facilitated walking intervention in increasing physical activity levels in people with LBP.

**METHODS**

**Design**

The study design will be a randomised controlled trial. Participants will be randomised into two groups; Group 1/Experimental Group/Intervention Group: usual physiotherapy care plus Fitbit facilitated walking intervention and Group 2/Control Group: usual physiotherapy care alone. The study flowchart is displayed in **Figure 1**. This protocol was developed to adhere with the guidelines of the Standard Protocol Items: Recommendations for Interventional Trials (SPIRIT) and will be reported to comply with the 25-item Consolidated Standards of Reporting Trials(CONSORT) statement on trial reporting.^30^

Follow-up (week 26)

Participants with low back pain attending the physiotherapy clinic will be informed by the administrative staff about the study

Allocated to the experimental group (n=34)

Excluded

Inclusion criteria not met

Allocated to the control group (n= 34)

## Enrolment

Declines to participate

Screening: participants will be assessed for eligibility

Informed consent

Baseline data collection

Follow-up (week 26)

Post-intervention (week 9)

Post-intervention (week 9)

## Randomisation

## Post-intervention

## Follow-up

**Figure 1** Flow of participants through the trial.

**Eligibility criteria for participants**

Participants will be included in this study if they:

1. are diagnosed by physiotherapist with non-specific LBP;
2. are aged 18 years or over;
3. are categorized as being at medium or high risk of chronicity using STarT Back Screening Tool^31^;
4. are classified as physically inactive (those who engage in less than 150 minutes/week of moderate intensity, or less than 75 minutes/week of vigorous intensity or an equivalent combination of the two intensities of physical activity as determined by International Physical Activity Questionnaire (IPAQ)^32^;
5. are ready and able to participate in physical activity as determined by The Physical Activity Readiness Questionnaire (PAR-Q).^33^ Those deemed not fit to participate in physical activity by the PAR-Q or aged over 69 years, will need a clearance from their medical practitioner before engaging in physical activity;
6. have internet access.

Participants will be excluded if they have any:

1. contraindications to physical exercise (cardiovascular diseases e.g. myocardial infarction, embolism, or uncontrolled diabetes; orthopaedic impairments; balance problems);
2. serious spinal pathologies (e.g. fractures, tumours or inflammatory diseases such as ankylosing spondylitis);
3. neurological compromise (e.g. spinal nerve compromise or cauda equina syndrome);
4. pregnancy.

**Sample size**

A sample size was calculated using a similar method to that of a published study^34^ with a similar design which reported a mean post intervention disability score of 15.8 (SD=12.7) (measured by Oswestry LBP Disability Questionnaire) in the experimental group and 30.6 (SD=16.9) in the control group. The aim is to achieve 90% power to observe a significant difference of 5% (alpha of 0.05) using a two-tailed t-test. To allow for a 20% drop-out, the sample size was estimated to be 68 participants (34 participants in each group).

**Setting and recruitment**

Participants will be recruited from the physiotherapy outpatient departments of public and private hospitals or private physiotherapy practices in Sydney, Australia. Participants referred to the clinic will be informed about the study by the administrative staff who will also seek the approval of potential participants to pass their contact details to the associate investigator. Recruitment flyers will also be left in the waiting room or will be passed out by the staff to interested participants visiting the clinics. The investigator will contact participants via email and/or phone and provide more details about the study, check for the eligibility criteria and administer the surveys. A meeting will be arranged with the participating physiotherapists to explain the study protocol and agree on the research procedures.

**Enrolment**

Interested people with LBP will be assessed for eligibility to participate in the study by the therapist and investigator. Eligible people will receive information about the study protocol and written informed consent will be obtained. Also, the investigator will explain the protocol verbally and answer the participants’ questions before obtaining written informed consent. Participants who will sign the informed consent will be included in this study.

**Randomisation**

After confirmation of eligibility and signed informed consent is obtained, baseline outcome measurements will be collected (**Table 1**). After that, participants will be randomly allocated to one of the two groups. The randomisation process will be performed using computer-generated random numbers. The allocation sequence will be concealed from the investigator screening participants for inclusion and contained in sequentially, numbered, opaque and sealed envelopes. Usual physiotherapy care will be provided to participants by their treating physiotherapists who will determine the treatment pragmatically based on clinical reasoning. Participants may receive different treatment modalities depending on their physiotherapist and clinical presentation. Therefore, stratified block randomisation will be used to ensure fidelity and balance of the usual physiotherapy care given to participants in both groups at each participating clinic, and also to ensure equal sample sizes.

**Interventions**

**Control Group**

Participants allocated in the control group will receive usual physiotherapy care provided by their treating physiotherapist. Also, they will be given instructions to maintain their usual physical activity level during the treatment period.

**Experimental Group**

Participants in the experimental group will receive a Fitbit walking intervention provided by the investigators in addition to usual physiotherapy care provided by their treating physiotherapist.

During the baseline period, participants will be requested to wear a physical activity monitor (Axivity AX3 and Fitbit Flex wearable devices) for seven continuous days, to measure the total number of habitual walking steps per week. The baseline average daily walking steps for participants will be calculated by dividing the total number of habitual walking steps per week by seven (total walking steps per week/7). During the intervention each participant’s average daily walking steps will be progressed each week by a minimum 10%. To comply with current physical activity guidelines, participants will be asked to walk the prescribed number of steps at a moderate intensity (i.e. brisk walking, 100 step/min).^35-38^ Participants will be asked to partake in the prescribed walking program at least five days per week for 8 weeks.

Participants in the experimental group will receive a Fitbit Flex wearable device and will attend a training session delivered by the investigator to: 1) install and set up the Fitbit wearable device and account, 2) register the participant in the 10,000 Steps website and synchronise the device with the Fitbit account, and 3) provided with instructions of using the device and account including monitoring step counts as well as other features. Additionally, participants will receive a handbook detailing all of the instructions provided from the training session.

**“Fitbit Facilitated Walking Intervention” conceptual basis and development:**

The Fitbit facilitated walking intervention was developed for patients with LBP, using physical activity guidelines^39, 40^ in order to assist them maintain their usual activities, and to improve their compliance with the prescribed therapeutic exercise. The intervention consists of: 1) Fitbit wearable device, 2) 10,000 Steps website, and 3) progressive walking program.

The Fitbit device will enable participants to monitor their progress in meeting the target number of steps. Further, the Fitbit wearable device will act as a motivational feedback tool providing immediate information on activity levels. The 10,000 Steps website is a non-profit promotion web-based platform that was developed to increase the physical activity level of the general community and it can be connected and synchronised with the Fitbit account. We are using this website to encourage participants to use the Fitbit device, track and monitor the daily steps progress and share it with other participants. Also, it enables the investigator to monitor and track the participants’ progress and keep in contact with them. The website has high levels of usability.^41^

The average steps inactive people walk (for any purpose) is less than 5000 steps per day.^35, 36^ Therefore, for people to adhere to PA guidelines, they need to start a graded walking program which will be progressed by at least 10% each week.

**Participant’s adherence to intervention and motivation:**

1. Participants’ and investigator’s accounts will be connected using the “Fitbit friends” feature, which allows for Fitbit users to share and compare stats with each other for motivation.
2. The data will be synced periodically to the Fitbit account from the wearable Fitbit device. The Fitbit application uses a Bluetooth Low Energy technology to sync with the Fitbit wearable device. Every time the participant opens the application, the data will be synced if the wearable device is nearby. However, if the compute does not have a Bluetooth, the participants will be instructed to plug the wireless sync dongle into the computer.
3. The participants’ data will be monitored by the investigator using the 10,000 Steps website ([www.10000steps.org.au](http://www.10000steps.org.au)).^42^
4. Participants can log in to the Fitbit account and the 10,000 Steps website and track their progress, and contact the investigator via the 10,000 Steps website.
5. The investigator will have the list of participant’s names with their information so that he/she can track the patients’ adherence and progress, and provide feedback by email, text message or telephone call during the treatment period every week.
6. Participants will receive daily motivational messages from the Fitbit wearable device.

Adherence to the walking intervention will be measured using the Fitbit device by assessing the duration of walking, and number of steps attained, and so intensity of walking (walking steps/walking duration).

**Measures**

**Sociodemographic data**

At the initial assessment demographic information including age, gender, employment status, level of education, health condition, body mass index, smoking and LBP history (previous episodes of LBP, previous surgery for LBP and previous LBP treatments received) will be collected.

**STarT Back Screening Tool**

The STarT Back Screening Tool^31^ has been designed to help clinicians categorise participants with LBP into low, medium or high risk according to their risk of chronicity, developing persistent disabling pain. It is composed of 9 questions about distribution of pain, disability, fear, anxiety, pessimistic thoughts, depression and bothersomeness in the last two weeks. Participants will be asked to choose either agree (0 point) or disagree (1 point) for each question except Question 9 which uses Likert scale comprising 5 possible options (Not at all=0, slightly=0, moderately=0, very much=1 and extremely=1). The total score (1-9) and the psychosocial sub score (5-9) will be calculated. A Score ≤3 point (≤3/9) indicates low risk. Scores ≥4 points (≥4/9) but ≤3 points on the psychosocial sub score **(**≤3/5) indicates medium risk. Scores ≥4 points (≥4/9) but ≥4 points on the psychosocial sub score **(**≥4 /5) indicates high risk. Therefore participants with a score above 3 will be eligible to join the study. The STarT Back Screening Tool has been shown to be valid in identifying subgroups of participants with LBP.^31^

**Fitbit Flex wearable device**

The physical activity level and average number of walking steps per day for participants in the experimental group will be measured by Fitbit Flex wearable device.^43^ The Fitbit Flex is a wristband activity monitor that contains a tri-axial accelerometer. The data can be uploaded to the Fitbit website which will be synchronized with 10,000 Steps website. This device has been shown to be accurate and valid in measuring physical activity and quantifying steps.^27, 28, 44^

**Walking intervention adherence**

Participants’ adherence to the walking intervention will be determined using the 10,000 Steps Website and Fitbit Flex device during the treatment period (8 weeks).

**Primary outcomes**

**Disability**

Disability will be measured by the modified Oswestry Disability Index (ODI).^45^ The modified ODI is an important tool used by researchers and clinicians in measuring functional disability in participants with LBP. It is a self-administered questionnaire consisting of ten sections designed to evaluate the activities of daily living. Each section contains six statements scored from 0 (minimum difficulty in performing activity) to 5 (maximum difficulty). The total score ranges from 0 (no disability) to 50 (greatest disability). The modified ODI is valid, reliable and responsive to change in disability in participants with LBP.^45, 46^

**Pain**

Pain intensity will be measured by the Visual Analogue Scale (VAS).^47^ The VAS is one of the most commonly used scales in measuring pain intensity, providing a numerical rating ranging from 0 (no pain) to 10 (worst pain). The VAS has been shown to be valid and reliable for assessing pain in participants with LBP.^48^

**Secondary outcomes**

**Habitual physical activity level and walking steps**

Habitual physical activity level and walking steps in both groups will be measured objectively by Axivity AX3 (Newcastle upon Tyne, UK; product website: http://axivity.com/product/ax3) for seven continuous days. The Axivity AX3 is a wristband activity monitor that contains a tri-axial accelerometer. The device is waterproof, small, light and is ideal for collecting longitudinal movement data. This device has been shown to be accurate and valid in measuring walking steps.^49-51^

**Depression**

Depression will be measured by Beck Depression Inventory (BDI).^52^ It is one of the most commonly used psychological tests for assessing the severity of depression. The BDI is a 21-item self-administered questionnaire; each item contains 4 statements ranging from 0 to 3 based on severity of each item. A higher total score (29-63) indicates severe depression. This tool has a good internal consistency and good content validity.^53^

**Pain catastrophising**

Participants’ experience of pain will be measured by Pain Catastrophising Scale (PCS).^54^ The PCS is a 13-item self-administered questionnaire, each item consists of a 5-point scale ranging from 0 (not at all) to 4 (all the time). The PCS has been shown to be reliable and valid.^55, 56^

**Fear of movement**

Fear of movement will be measured by Tampa Scale for Kinesiophobia (TSK).^57^ This tool is widely used in clinical practice to assess the fear of movement resulting from feeling of vulnerability to re-injury in participants with pain. It is a 17-item self-administered questionnaire, with four-point rating scale (1=strongly disagree, 2=disagree, 3=agree and 4=strongly agree). The TSK has a good validity and reliability.^58, 59^

The primary and secondary outcomes measurement tools and time-points are described in **Table 1**.

| Table 1 Outcome measurement tools and time-points | | | |
| --- | --- | --- | --- |
|  | **Outcome** | **Measurement tool** | **Measurement points** |
| *Primary outcomes* | Disability | Oswestry LBP Disability Questionnaire^60^ | Baseline, post-intervention, follow-up (week 26) |
|  | Pain | Visual Analogue Scale^47^ | Baseline, post-intervention, follow-up (week 26) |
| *Secondary outcomes* | Habitual physical activity level + Habitual daily walking steps | Axivity AX3 | Baseline, post-intervention, follow-up (week 26) |
|  | Depression | Beck Depression Inventory II^61^ | Baseline, post-intervention, follow-up (week 26) |
|  | Fear of movement | Tampa Scale for Kinesiophobia^57^ | Baseline, post-intervention, follow-up (week 26) |
|  | Pain catastrophising | Pain Catastrophising Scale^54^ | Baseline, post-intervention, follow-up (week 26) |

**Study timeline**

The duration of the intervention will be 8 weeks. The outcomes will be assessed at baseline (week 0), post intervention (week 9) and post-randomisation (week 26). See **Table 1** for more details about the measurement tools and times

**Safety and adverse events**

Walking programs are generally safe,^62^ however, it is possible that participants may experience some unforeseen problems such as muscle soreness, falls or other injuries. Participants will be closely monitored to keep track of any unwanted effects or any problems. If there are any unwanted effects the program may be stopped and the reasons will be discussed with the treating physiotherapist and investigator. In case of a serious adverse event, participants will be advised to seek immediate medical assistance.

**Data collection and management**

The subjective data (disability, pain, depression, fear of movement and pain catastrophising) will be collected online using Research Electronic Data Capture (REDCap).^63^ The objective data (habitual physical activity level and daily walking steps) will be collected by Axivity AX3**.**

**Statistical analysis**

Data will be analyzed using Statistical Package for Social Sciences (SPSS) version 22. Sociodemographic variables, physical activity and clinical characteristics will be described by means, standard deviations and frequencies. Generalized linear models will be used to evaluate the effect of the treatment on the primary and secondary outcomes. The statistical analysis will follow the intention-to-treat principle in which all participants will be analysed in the groups to which they were randomised, regardless of whether they withdraw from their allocation.^64^ However, if participants withdraw from their randomised group, attempts will be made to obtain permission to collect outcome data at the follow-up time points. The statistical analysis will be conducted by an investigator who will be blinded to the subject’s intervention group.

**DISCUSSION**

We have presented the rationale and design of this trial. This trial will investigate the effect of using a Fitbit device to facilitate a walking intervention in addition to usual care provided by a physiotherapist. We will be able to determine the effect of progressive walking in management LBP.

We have developed the Fitbit facilitated walking intervention program to improve the patients’ adherence to the graded walking program and then increasing the physical activity level. Results from this study will inform clinical practice by providing evidence about the effectiveness of this program in reducing disability and pain, increasing physical activity levels, and improving functional and psychological status in participants with LBP.

REFERENCESUncategorized References

1. Hoy D, Bain C, Williams G, March L, Brooks P, Blyth F, et al. A systematic review of the global prevalence of low back pain. *Arthritis and rheumatism.* 2012;64(6): 2028-2037.

2. Walker BF. The Prevalence of Low Back Pain: A Systematic Review of the Literature from 1966 to 1998. *Journal of Spinal Disorders.* 2000;13(3): 205-217.

3. Airaksinen O, Brox JI, Cedraschi C, Hildebrandt J, Klaber-Moffett J, Kovacs F, et al. Chapter 4 European guidelines for the management of chronic nonspecific low back pain. *Eur Spine J.* 2006;15(S2): s192-s300.

4. Balague F, Mannion AF, Pellise F, Cedraschi C. Non-specific low back pain. *Lancet.* 2012;379(9814): 482-491.

5. Leung L. Pain Catastrophizing: An Updated Review. *Indian Journal of Psychological Medicine.* 2012;34(3): 204-217.

6. Valat JP. Factors involved in progression to chronicity of mechanical low back pain. *Joint Bone Spine.* 2005;72(3): 193-195.

7. Waddell G, Newton M, Henderson I, Somerville D, Main CJ. A Fear-Avoidance Beliefs Questionnaire (FABQ) and the role of fear-avoidance beliefs in chronic low back pain and disability. *Pain.* 1993;52(2): 157-168.

8. van Tulder M, Koes B, Bombardier C. Low back pain. *Best practice & research Clinical rheumatology.* 2002;16(5): 761-775.

9. Stefane T, Amanda Munari dos S, Marinovic A, Hortense P. Chronic low back pain: pain intensity, disability and quality of life/Dor lombar crônica: intensidade de dor, incapacidade e qualidade de vida. *Acta Paulista de Enfermagem.* 2013;26(1): 14.

10. Hoy D, Brooks P, Blyth F, Buchbinder R. The Epidemiology of low back pain. *Best Practice & Research Clinical Rheumatology.* 2010;24(6): 769-781.

11. Meucci RD, Fassa AG, Faria NMX. Prevalence of chronic low back pain: systematic review. *Revista de saude publica.* 2015;49.

12. Maniadakis N, Gray A. The economic burden of back pain in the UK. *Pain.* 2000;84(1): 95-103.

13. Westman AE, Boersma K, Leppert J, Linton SJ. Fear-Avoidance Beliefs, Catastrophizing, and Distress: A Longitudinal Subgroup Analysis on Patients With Musculoskeletal Pain. *The Clinical journal of pain.* 2011;27(7): 567-577.

14. Wideman TH, Sullivan MJ. Development of a cumulative psychosocial factor index for problematic recovery following work-related musculoskeletal injuries. *Phys Ther.* 2012;92(1): 58-68.

15. Smeets RJEM, Vlaeyen JWS, Kester ADM, Knottnerus JA. Reduction of Pain Catastrophizing Mediates the Outcome of Both Physical and Cognitive-Behavioral Treatment in Chronic Low Back Pain. *The Journal of Pain.* 2006;7(4): 261-271.

16. Marshall PWM, Schabrun S, Knox MF. Physical activity and the mediating effect of fear, depression, anxiety, and catastrophizing on pain related disability in people with chronic low back pain. *PLoS ONE.* 2017;12(7): e0180788.

17. Verbunt JA, Seelen HA, Vlaeyen JW, van der Heijden GJ, Knottnerus JA. Fear of injury and physical deconditioning in patients with chronic low back pain<sup>1</sup>. *Archives of Physical Medicine and Rehabilitation.*84(8): 1227-1232.

18. Vlaeyen JWS, Kole-Snijders AMJ, Boeren RGB, van Eek H. Fear of movement/(re)injury in chronic low back pain and its relation to behavioral performance. *Pain.* 1995;62(3): 363-372.

19. Lin C-WC, McAuley JH, Macedo L, Barnett DC, Smeets RJ, Verbunt JA. Relationship between physical activity and disability in low back pain: A systematic review and meta-analysis. *PAIN®.* 2011;152(3): 607-613.

20. Warburton DER, Nicol CW, Bredin SSD. Health benefits of physical activity: the evidence. *CMAJ : Canadian Medical Association Journal.* 2006;174(6): 801-809.

21. Siegel PZ, Brackbill RM, Heath GW. The epidemiology of walking for exercise: implications for promoting activity among sedentary groups. *Am J Public Health.* 1995;85(5): 706-710.

22. Krein SL, Kadri R, Hughes M, Kerr EA, Piette JD, Holleman R, et al. Pedometer-based internet-mediated intervention for adults with chronic low back pain: randomized controlled trial. *Journal of Medical Internet Research.* 2013;15(8): e181.

23. Torstensen TA, Ljunggren AE, Meen HD, Odland E, Mowinckel P, Geijerstam SA. Efficiency and costs of medical exercise therapy, conventional physiotherapy, and self-exercise in patients with chronic low back pain: A pragmatic, randomized, single-blinded, controlled trial with 1-year follow- up. *Spine.* 1998;23(23): 2616-2624.

24. Sallis R, Franklin B, Joy L, Ross R, Sabgir D, Stone J. Strategies for Promoting Physical Activity in Clinical Practice. *Progress in Cardiovascular Diseases.* 2015;57(4): 375-386.

25. Bravata DM, Smith-Spangler C, Sundaram V, Gienger AL, Lin N, Lewis R, et al. Using pedometers to increase physical activity and improve health: a systematic review. *Jama.* 2007;298(19): 2296-2304.

26. Ogilvie D, Foster CE, Rothnie H, Cavill N, Hamilton V, Fitzsimons CF, et al. Interventions to Promote Walking: Systematic Review. *BMJ: British Medical Journal.* 2007;334(7605): 1204-1207.

27. Diaz KM, Krupka DJ, Chang MJ, Peacock J, Ma Y, Goldsmith J, et al. FITBIT(®): AN ACCURATE AND RELIABLE DEVICE FOR WIRELESS PHYSICAL ACTIVITY TRACKING. *International journal of cardiology.* 2015;185: 138-140.

28. Evenson KR, Goto MM, Furberg RD. Systematic review of the validity and reliability of consumer-wearable activity trackers. *The International Journal of Behavioral Nutrition and Physical Activity.* 2015;12: 159.

29. Cadmus-Bertram L, Marcus BH, Patterson RE, Parker BA, Morey BL. Use of the Fitbit to Measure Adherence to a Physical Activity Intervention Among Overweight or Obese, Postmenopausal Women: Self-Monitoring Trajectory During 16 Weeks. *JMIR mHealth and uHealth.* 2015;3(4): e96.

30. Schulz KF, Altman DG, Moher D, Group C. Consort 2010 statement: Updated guidelines for reporting parallel group randomized trials. *Ann Intern Med.* 2010;152(11): 726-732.

31. Hill JC, Dunn KM, Lewis M, Mullis R, Main CJ, Foster NE, et al. A primary care back pain screening tool: identifying patient subgroups for initial treatment. *Arthritis and rheumatism.* 2008;59(5): 632-641.

32. Craig CL, Marshall AL, Sjostrom M, Bauman AE, Booth ML, Ainsworth BE, et al. International physical activity questionnaire: 12-country reliability and validity. *Medicine and science in sports and exercise.* 2003;35(8): 1381-1395.

33. Canadian Society for Exercise Physiology. Physical Activity and Readiness Questionnaire: PAR-q & You; 2002. <http://www.csep.ca/cmfiles/publications/parq/par-q.pdf>.

34. Murtezani A, Hundozi H, Orovcanec N, Sllamniku S, Osmani T. A comparison of high intensity aerobic exercise and passive modalities for the treatment of workers with chronic low back pain: a randomized, controlled trial. *Eur J Phys Rehabil Med.* 2011;47(3): 359-366.

35. Tudor-Locke C, Hatano Y, Pangrazi RP, Kang M. Revisiting "how many steps are enough?". *Medicine and science in sports and exercise.* 2008;40(7 Suppl): S537-S543.

36. Tudor-Locke C, Bassett DR. How Many Steps/Day Are Enough? *Sports Med.* 2004;34(1): 1-8.

37. Abel M, Hannon J, Mullineaux D, Beighle A. Determination of step rate thresholds corresponding to physical activity intensity classifications in adults. *J Phys Act Health.* 2011;8(1): 45-51.

38. Rowe DA, Welk GJ, Heil DP, Mahar MT, Kemble CD, Calabrá MA, et al. Stride rate recommendations for moderate-intensity walking. *Medicine and science in sports and exercise.* 2011;43(2): 312-318.

39. Koes BW, van Tulder M, Lin C-WC, Macedo LG, McAuley J, Maher C. An updated overview of clinical guidelines for the management of non-specific low back pain in primary care. *Eur Spine J.* 2010;19(12): 2075-2094.

40. National Institute for Health and Clinical Excellence. Low Back Pain and Sciatica in Over 16s: Assessment and Management. 2016.

41. Davies C, Joyner K, Van Itallie A, Mummery K. Usefulness and usability of the 10,000 steps website within a sample of current step log users. *J Sci Med Sport.* 2010;12: e151-e152.

42. CQUniversity Australia. 10,000 Steps Website; 2016. <https://www.10000steps.org.au/>.

43. Fitbit. Make Fitness a Lifestyle with Flex; <https://www.fitbit.com/au/flex>.

44. Sushames A, Edwards A, Thompson F, McDermott R, Gebel K. Validity and Reliability of Fitbit Flex for Step Count, Moderate to Vigorous Physical Activity and Activity Energy Expenditure. *PLoS ONE.* 2016;11(9): e0161224.

45. Fritz JM, Irrgang JJ. A comparison of a modified Oswestry Low Back Pain Disability Questionnaire and the Quebec Back Pain Disability Scale. *Phys Ther.* 2001;81(2): 776-788.

46. Haas M, Jacobs GE, Raphael R, Petzing K. Low back pain outcome measurement assessment in chiropractic teaching clinics: responsiveness and applicability of two functional disability questionnaires. *Journal of manipulative and physiological therapeutics.* 1995;18(2): 79-87.

47. Gould D, Kelly D, Goldstone L, Gammon J. Examining the validity of pressure ulcer risk assessment scales: developing and using illustrated patient simulations to collect the data. *Journal of clinical nursing.* 2001;10(5): 697-706.

48. Olaogun MOB, Adedoyin RA, Ikem IC, Anifaloba OR. Reliability of rating low back pain with a visual analogue scale and a semantic differential scale. *Physiotherapy Theory and Practice.* 2004;20(2): 135-142.

49. Feng Y, Wong CK, Janeja V, Kuber R, Mentis HM. Comparison of tri-axial accelerometers step-count accuracy in slow walking conditions. *Gait & Posture.* 2017;53: 11-16.

50. Aodhán H, Silvia Del D, Lynn R, Alan G. Detecting free-living steps and walking bouts: validating an algorithm for macro gait analysis. *Physiological Measurement.* 2017;38(1): N1.

51. Godfrey A, Del Din S, Barry G, Mathers JC, Rochester L. Within trial validation and reliability of a single tri-axial accelerometer for gait assessment. *Conference proceedings : Annual International Conference of the IEEE Engineering in Medicine and Biology Society IEEE Engineering in Medicine and Biology Society Annual Conference.* 2014;2014: 5892-5895.

52. Beck AT, Ward CH, Mendelson M, Mock J, Erbaugh J. An inventory for measuring depression. *Archives of general psychiatry.* 1961;4: 561-571.

53. Richter P, Werner J, Heerlein A, Kraus A, Sauer H. On the validity of the Beck Depression Inventory. A review. *Psychopathology.* 1998;31(3): 160-168.

54. Sullivan MJL, Bishop SR, Pivik J. The Pain Catastrophizing Scale: Development and validation. *Psychological Assessment.* 1995;7(4): 524-532.

55. Osman A, Barrios FX, Kopper BA, Hauptmann W, Jones J, O'Neill E. Factor structure, reliability, and validity of the Pain Catastrophizing Scale. *Journal of behavioral medicine.* 1997;20(6): 589-605.

56. Osman A, Barrios FX, Gutierrez PM, Kopper BA, Merrifield T, Grittmann L. The Pain Catastrophizing Scale: further psychometric evaluation with adult samples. *Journal of behavioral medicine.* 2000;23(4): 351-365.

57. Miller RP, Kori S, D T. The Tampa Scale for Kinesiophobia. *The Clinical journal of pain.* 1991;7(1): 51-52.

58. Lundberg MKE, Styf J, Carlsson SG. A psychometric evaluation of the Tampa Scale for Kinesiophobia — from a physiotherapeutic perspective. *Physiotherapy Theory and Practice.* 2004;20(2): 121-133.

59. Vlaeyen JW, Kole-Snijders AM, Boeren RG, van Eek H. Fear of movement/(re)injury in chronic low back pain and its relation to behavioral performance. *Pain.* 1995;62(3): 363-372.

60. Fairbank JC, Couper J, Davies JB, O'Brien JP. The Oswestry low back pain disability questionnaire. *Physiotherapy.* 1980;66(8): 271-273.

61. Beck AT, Steer RA, Brown GK. *BDI*: Ediciones Paidos Iberica, S.A.; 2006.

62. Physical Activity Guidelines Advisory Committee. Physical activity guidelines advisory committee report. *Washington DC: US Department of Health and Human Services.* Chapter 6. US Department of Health and Human Services; 2008.

63. Obeid JS, McGraw CA, Minor BL, Conde JG, Pawluk R, Lin M, et al. Procurement of shared data instruments for Research Electronic Data Capture (REDCap). *Journal of Biomedical Informatics.* 2013;46(2): 259-265.

64. Gupta SK. Intention-to-treat concept: A review. *Perspectives in Clinical Research.* 2011;2(3): 109-112.
